# Mutational activation of BRAF confers sensitivity to transforming growth factor beta inhibitors in human cancer cells

**DOI:** 10.18632/oncotarget.13226

**Published:** 2016-11-09

**Authors:** Lindsay C. Spender, G. John Ferguson, Sijia Liu, Chao Cui, Maria Romina Girotti, Gary Sibbet, Ellen B. Higgs, Morven K. Shuttleworth, Tom Hamilton, Paul Lorigan, Michael Weller, David F. Vincent, Owen J. Sansom, Margaret Frame, Peter ten Dijke, Richard Marais, Gareth J. Inman

**Affiliations:** ^1^ Growth Factor Signalling Laboratory, The Beatson Institute for Cancer Research, Bearsden, Glasgow, United Kingdom; ^2^ Biological Services, The Beatson Institute for Cancer Research, Bearsden, Glasgow, United Kingdom; ^3^ Colorectal Cancer and Wnt Signalling, The Beatson Institute for Cancer Research, Bearsden, Glasgow, United Kingdom; ^4^ Department of Molecular Cell Biology, Cancer Genomics Centre Netherlands, Leiden University Medical Center, Einthovenweg, Leiden, Netherlands; ^5^ Cancer Research UK Manchester Institute, The University of Manchester, Wilmslow Road, Withington, Manchester, United Kingdom; ^6^ The University of Manchester, The Christie NHS Foundation Trust, Manchester, United Kingdom; ^7^ Department of Neurology, University Hospital Zurich, Frauenklinikstrasse, Zurich, Switzerland; ^8^ The Institute of Genetics and Molecular Medicine, Edinburgh Cancer Research Centre, University of Edinburgh, Western General Hospital, Edinburgh, United Kingdom; ^9^ Division of Cancer Research, School of Medicine, University of Dundee, Dundee, United Kingdom; ^10^ Department of Respiratory, Inflammation and Autoimmunity Research, MedImmune Limited, Cambridge, United Kingdom

**Keywords:** melanoma, BRAF, vemurafenib, PLX-4720, TGF-beta

## Abstract

Recent data implicate elevated transforming growth factor-β (TGFβ) signalling in BRAF inhibitor drug-resistance mechanisms, but the potential for targeting TGFβ signalling in cases of advanced melanoma has not been investigated. We show that mutant BRAFV600E confers an intrinsic dependence on TGFβ/TGFβ receptor 1 (TGFBR1) signalling for clonogenicity of murine melanocytes. Pharmacological inhibition of the TGFBR1 blocked the clonogenicity of human mutant BRAF melanoma cells through SMAD4-independent inhibition of mitosis, and also inhibited metastasis in xenografted zebrafish. When investigating the therapeutic potential of combining inhibitors of mutant BRAF and TGFBR1, we noted that unexpectedly, low-dose PLX-4720 (a vemurafenib analogue) promoted proliferation of drug-naïve melanoma cells. Pharmacological or pharmacogenetic inhibition of TGFBR1 blocked growth promotion and phosphorylation of SRC, which is frequently associated with vemurafenib-resistance mechanisms. Importantly, vemurafenib-resistant patient derived cells retained sensitivity to TGFBR1 inhibition, suggesting that TGFBR1 could be targeted therapeutically to combat the development of vemurafenib drug-resistance.

## INTRODUCTION

Malignant melanoma is the most aggressive form of skin cancer with around 55,500 deaths worldwide in 2012 [1]. While primary localised melanoma may be cured by surgical removal alone, metastatic melanoma is associated with poor long-term prognosis. Somatic mutations that constitutively activate the RAS-RAF-mitogen activated protein kinase-extracellular signal-regulated kinase (RAS-RAF-MEK-ERK) signalling pathway are frequently detected in melanoma; mutations in BRAF and NRAS have been detected in approximately 50% and 20% of melanomas, respectively [2]. The identification of genetic drivers of melanoma [2] has led to the development of small-molecule inhibitors inhibitors (e.g. vemurafenib, dabrafenib) (BRAFi), which selectively target mutant BRAF. Their use in the clinic has significantly increased survival of metastatic melanoma patients [3–5]. However, the development of drug resistance remains a significant problem with the vast majority of patients with advanced melanoma dying of drug-resistant disease.

Numerous mechanisms of resistance to BRAF inhibitors have been described, many involving reactivation of the MAPK pathway (reviewed in [6]). As a result, the combined use of BRAF inhibitors with MEK inhibitors (e.g. cobimetinib, trametinib) has been proposed as a way to overcome the development of resistance [7–9]. While this approach significantly improves patient survival (resulting in a median expected survival of approximately 25 months for eligible patients), the efficacy of combinatorial therapies which target the same signalling pathway ultimately may be limited because of augmented BRAF inhibitor drug resistance mechanisms or secondary mutations [10, 11].

Additionally, secondary epigenetic events that do not necessarily affect MEK/ERK activity can occur to limit the tumour cells' dependence on the MAPK pathway, or restrict tumour immune surveillance. These resistance mechanisms include changes in the methylome affecting tumour cell apoptosis [12], increases in PI3K/AKT activity [13–15] and/or increases in receptor tyrosine kinase (RTK) signalling. For instance, loss of microphthalmia-associated transcription factor (MITF) expression correlates with increased RTK expression and resistance [16]. Vemurafenib-resistance induced increases in EGFR signalling have been shown to activate an EGFR-SRC-STAT3 signalling cascade in melanoma, and targeting this pathway using inhibitors of SRC inhibits growth of vemurafenib-resistant xenografts [17, 18].

As well as cell autonomous effects, drug-induced stimulation of melanoma-associated fibroblasts stimulates matrix remodelling and, in this case, signals *via* integrins to increase SRC and FAK activity. This change in the microenvironment promotes melanoma cell survival and provides a “safe haven” to enable emergence of drug-resistant tumour cells [19]. Clearly, stromal remodelling and SRC activation have emerged as contributors to BRAF inhibitor resistance, and it is apparent that the therapy-induced secretome is key in driving resistance. Increased transforming growth factor-beta (TGFβ) secretion may be part of the therapy-induced secretome, and has been implicated in both *in vitro* derived drug resistance [20] and in vemurafenib-resistant patient material [21]. Increased TGFβ signalling can result in an upregulation of EGFR and PDGFR [21], positioning TGFβ signalling upstream of well described vemurafenib-resistance associated RTK pathways. Despite this, the potential for TGFβ pathway inhibitors in combating BRAF kinase inhibitor resistance has not been studied to date.

TGFβ ligand binds to the constitutively active high affinity type 2 serine/threonine kinase receptor TGFBR2 which trans-phosphorylates and activates TGFBR1. As part of the canonical signalling pathway, TGFBR1 phosphorylates and activates the intracellular signalling transcription factors SMAD2 and SMAD3, and following binding to SMAD4, the SMAD complex accumulates in the nucleus where it regulates target gene transcription. Additionally, TGFβ can signal *via* numerous non-canonical pathways including RHO/ROCK, MAPK, and PI3-Kinase (reviewed in [22]). In normal melanocytes, TGFβ inhibits proliferation and DNA synthesis and induces melanocyte stem cell quiescence, however, melanoma cells are able to evade the tumour suppressive effects of TGFβ. TGFβ levels are elevated in the plasma of melanoma patients (regardless of their exposure to BRAF inhibitors), and increases in expression are associated with progressive disease [23]. The mechanisms of growth arrest and their evasion by melanoma cells, however, have not been fully characterised and are likely to be multi-factorial (reviewed in [24]).

There is little evidence of mutation of TGFβ receptors in melanoma [25], so, it appears that with functional receptors and apparently intact SMAD function [26, 27], melanoma cells are able to evade growth suppressive effects of TGFβ while simultaneously utilising pro-tumourigenic functions of TGFβ. TGFβ signalling promotes migration of BRAF-transformed melanocytes in *in vitro* organotypic skin cultures [28] and is involved in metastasis of mouse melanoma cells to the bone through expression of tissue-specific genes known to promote bone osteolysis [26, 29]. In addition, melanoma cells engineered to over-express TGFβ exert paracrine effects on stromal fibroblasts whereby they secrete matrix components (including fibronectin, collagens, and tenascin) to promote melanoma tumour formation [30]. These observations are reminiscent of the vemurafenib-induced activation of melanoma-associated fibroblasts providing a “safe haven” for melanoma tumour cells, however, no link has been formally established between vemurafenib-induced fibroblast activation and TGFβ signalling.

In this study, we now provide evidence that melanoma cells are “hard-wired” to depend on autocrine TGFβ signalling through TGFBR1 for tumour establishment and clonogenicity. We show that the fundamental addiction of melanoma cells to TGFβ is: induced by the presence of mutant BRAF; mediated by a SMAD4-independent pathway; and correlates with TGFβ regulation of RHOA activity, thus providing support for the notion that non-canonical signalling pathways are key mediators of pro-tumourigenic TGFβ function in melanoma. Importantly, we also provide evidence that vemurafenib resistant patient-derived cells retain sensitivity to inhibitors of TGFBR1. TGFBR1 inhibitors block the enhanced proliferation of paradoxically activated PLX-4720 treated melanoma cells, and can be used to effectively inhibit metastatic melanoma in a zebrafish xenograft model.

## RESULTS

### Mutant BRAF confers TGFβ addiction

**Figure 1:**
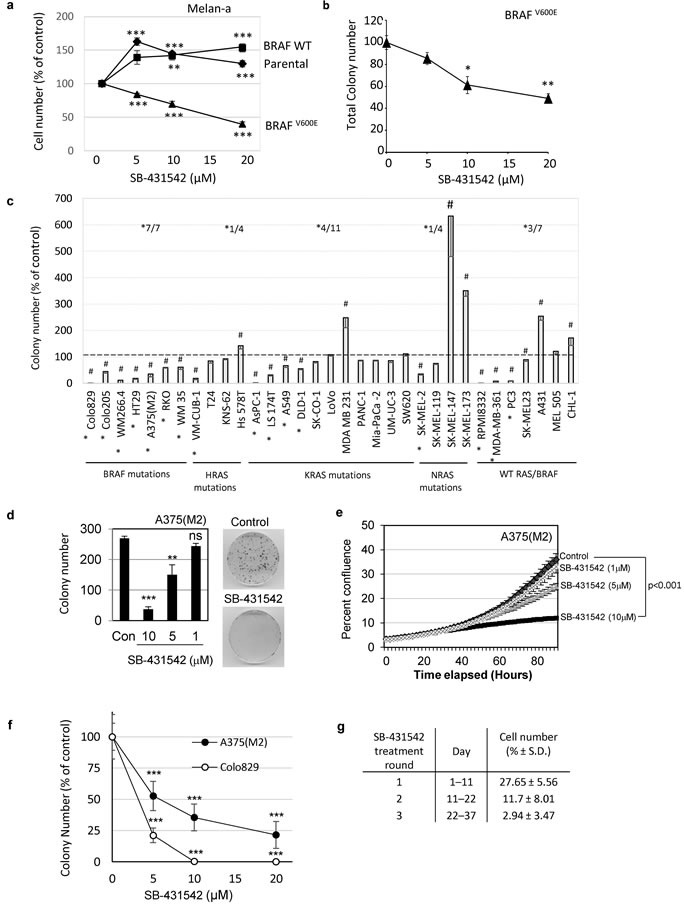
BRAF^V600E^ confers sensitivity to TGFBR1 inhibition **a.**, **b.** Melan-a cells expressing the indicated BRAF construct were seeded on plastic **a.**, or in soft agar for cells able to form anchorage independent colonies (mutant BRAF only) **b.**, in the presence of concentrations of SB-431542 as shown. The mean (± SEM) cell number after 6 days **a.** or colony number after 3-4 weeks **b**.were counted and presented as a percentage of the vehicle control. Data was pooled from *n* = 3-5 independent experiments each performed in triplicate. **c.** Cell lines with activating mutations in BRAF, HRAS, KRAS or NRAS or with wildtype BRAF/RAS were seeded in soft agar in the presence of 10μM SB-431542. Data is presented as a mean (± SD) colony number as a percentage of the vehicle control. Colony counts that were significantly different from controls following treatment with 10μM SB-431542 are indicated by (#) (TTEST, *p* < 0.05). SK-MEL-147 cells showed no colony formation in the presence of 0.2% DMSO and were assigned a value of 1 to allow analysis. The proportion of cell lines whose colony formation was inhibited by SB-431542 by more than 1/3 within each group is indicated on the histogram (*). **d.**-**g.** Mutant BRAF^V600E^ human melanoma cell lines A375(M2) (**d**-**g**) and Colo829 **f.** were treated with either vehicle control or the indicated concentrations of SB-431542. Colony growth on plastic **d.** or in soft agar **f.** were counted after 14 days and 4 weeks, respectively. **e.** Live cell imaging using an IncuCyte Zoom was used to determine the growth kinetics of A375(M2) cells seeded at low cell density (100 cells/well of 96-well plate) and treated with SB-431542. The mean percent confluence (± SEM) of 4 images per well (*n* = 24 from a representative experiment) is shown. Statistical analysis was carried out by pairwise comparison using the compareGrowthCurves function in statmod (R project). The adjusted p value (*p* < 0.001) is shown **g.** A375(M2) cells were serially passaged in the presence of 10μM SB-431542 to select resistant cells. Surviving cells after each round of treatment were reseeded at low cell density. Cell counts were determined at the end of each treatment round, and the results expressed as the mean (± SD) cell number from 6 wells as a percentage of the control (solvent control treated cells).

We demonstrated previously that autocrine signalling through TGFBR1, is required for transformation of rodent fibroblasts by oncogenic BRAF [31], but did not investigate this dependence in human models of activated RAS/RAF-driven cancer. Since mutational activation of BRAF is frequently observed in melanoma [2], we tested the susceptibility of immortalised mouse melanocytes stably transfected with either wild-type or mutant BRAF to inhibition by the TGFBR1 kinase inhibitor SB-431542. Unlike parental or wildtype BRAF transfected cells, melanocytes transfected with oncogenic V600E BRAF required TGFBR1 kinase activity for their proliferation since SB-431542 decreased cell numbers (Figure 1a). These data suggest that the presence of mutant BRAF in melanocytes confers a dependence (or addiction) on the TGFβ/TGFBR1 signalling pathway for cell proliferation. Similar results were observed in soft agar assays measuring anchorage independent growth (Figure 1b). We determined the amount of autocrine TGFβ produced by the transfected melanocytes, using a bioassay of NIH3T3 cells stably transfected with a CAGA_12_-luciferase reporter construct (Supplementary Figure 1a). The dependence on TGFBR1 activity for colony formation did not correlate simply with an increase in latent autocrine TGFβ production following transfection with mutant BRAF (no active TGFβ was detectable without medium acidification) (Supplementary Figure 1b). There was also no elevated signalling *via* the TGFβ receptor-regulated intracellular signalling transcription factor, SMAD2 in SB-431542 sensitive cells (Supplementary Figure 1c).

We tested whether human cancer cells with activating mutations in MAPK pathway components were also dependent on TGFBR1 for growth. A panel of human tumour cell lines carrying wild type RAS/BRAF or mutations in BRAF, HRAS, KRAS or NRAS (details of all cell lines are given in Supplementary Table 1) were tested for sensitivity to SB-431542 (Figure 1c). Inhibition of TGFBR1 resulted in a range of cellular responses in the wildtype, H-, K-, and N-RAS mutant groups, such that any dependence on TGFBR1 for colony formation could not be predicted in cells carrying these mutations. However, consistent with data obtained in mouse melanocytes (Figure 1b), colony formation in all seven human cell lines carrying mutant BRAF was significantly inhibited (Figure 1c). Again, sensitivity to the TGFBR1 inhibitor did not correlate with levels of autocrine TGFβ production (Supplementary Table S2). The effect of SB-431542 was dose-dependent in low density 2D-culture assay conditions established to assess more accurately clonogenic potential, reaching statistically significant inhibitory concentrations at 10μM (Figure 1d and 1e). Similar dose dependent effects were seen in anchorage-independent soft agar assays (Figure 1f). We attempted to select out TGFBR1 inhibitor-resistant cells following repeated rounds of treatment for over a month, but saw no evidence of outgrowth of refractory subpopulations or acquired-resistance during this time frame (Figure 1g). Taken together these data suggest that cells with mutational activation of BRAF, require TGFBR1 for efficient colony formation and that TGFβ would predictably function as a tumour promoter.

### Autocrine TGFβ is required for *in vivo* melanoma xenograft tumour formation

**Figure 2:**
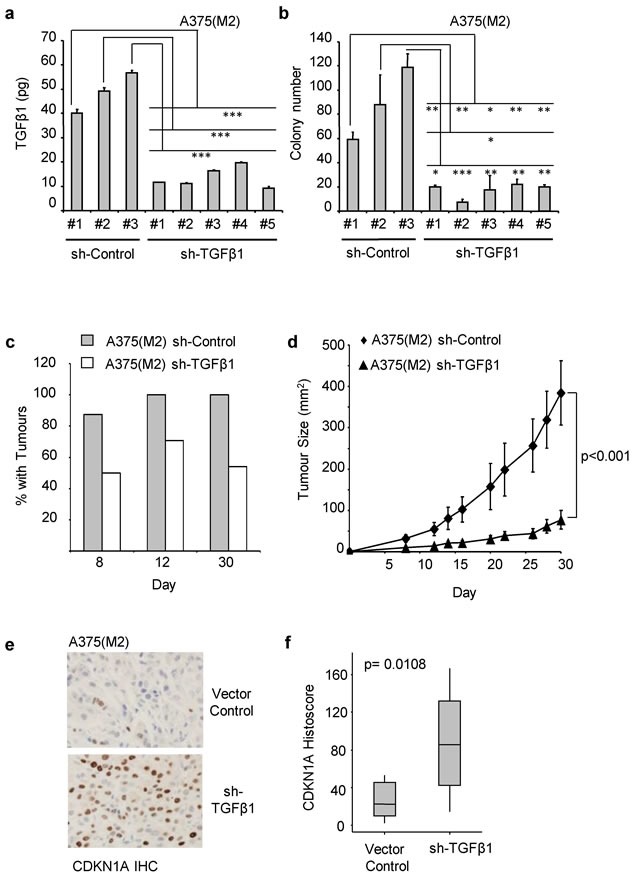
Colony and *in vivo* tumour formation require the autocrine production of TGFβ1 A375(M2) clones stably expressing a Control shRNA, or a TGFβ1 shRNA were analysed for TGFβ1 production **a.** and seeded into soft agar assays **b.**. **a.** TGFβ1 levels were analysed by ELISA and are expressed as the amount (pg) of TGFβ1 produced by 1x10^5^ cells/hour. Data shown are the means ± SD (*n* = 3). Colonies were counted and presented as the mean ± SD colony number (*n* = 3) **b.**. Statistical significance was measured using Students TTESTS (* = *p* < 0.05, ** = *p* < 0.01, *** = *p* < 0.001). (c-f) A375 (M2) clones, stably expressing either vector control or TGFβ1 shRNA were subcutaneously injected into the flanks of CD1 nude mice and tumours were allowed to develop. Palpable tumours were first detected after 8 days and were measured for a further 21 days. **c.** The number of mice (as a percentage of injected mice) that had palpable tumours on the indicated day (sh-Control, *n* = 16. sh-TGFβ1, *n* = 24). Statistical significance was measured using Students TTESTS (* = *p* < 0.05, ** = *p* < 0.01). **d.** Tumour volumes (mm^2^, mean ± SEM) were estimated on the indicated days post injection (sh-Control *n* = 16, sh-TGFβ1 *n* = 24). Statistical analysis was carried out using compareGrowthCurves (Statmod). **e.**, **f.** sh-control and sh-TGFβ1 tumour sections were stained for CDKN1A and counterstained with haematoxylin (sh-control, *n* = 8. sh-TGFβ1, *n* = 9). Representative images are shown in **e.** and the quantification of the resultant images by histoscore are shown in **f.**. The horizontal bar indicates the median histoscore, the grey boxes and vertical bars indicate 95% CI and range, respectively. P value following statistical analysis using Mann-Whitney U-test is shown.

Melanoma cells engineered to over-express TGFβ1 have increased tumour forming ability [30]. To discover whether endogenous autocrine TGFβ expression is required for tumour formation, we generated ligand knockdown clones of A375(M2) cells using stably transfected shRNA constructs targeting TGFβ1. Knockdown of TGFβ1 to levels below 20pg per 1x10^5^ cells/hr (Figure 2a) was sufficient to decrease the ability of A375(M2) cells to form colonies *in vitro* (Figure 2b and Supplementary Figure 2a). In xenograft assays, ligand knockdown reduced the percentage of mice with palpable tumours at all recorded time-points (Figure 2c), and significantly reduced tumour growth (Figure 2d). Immunohistochemical (IHC) staining of xenograft sections revealed increased expression of the cyclin-dependent kinase inhibitor CDKN1A (p21^CIP1^) in tumours generated by TGFβ knockdown cells (Figure 2e and 2f). Elevated CDKN1A^CIP1^ expression was also observed following SB-431542 treatment of A375(M2) cells (Supplementary Figure 2b).

**Figure 3:**
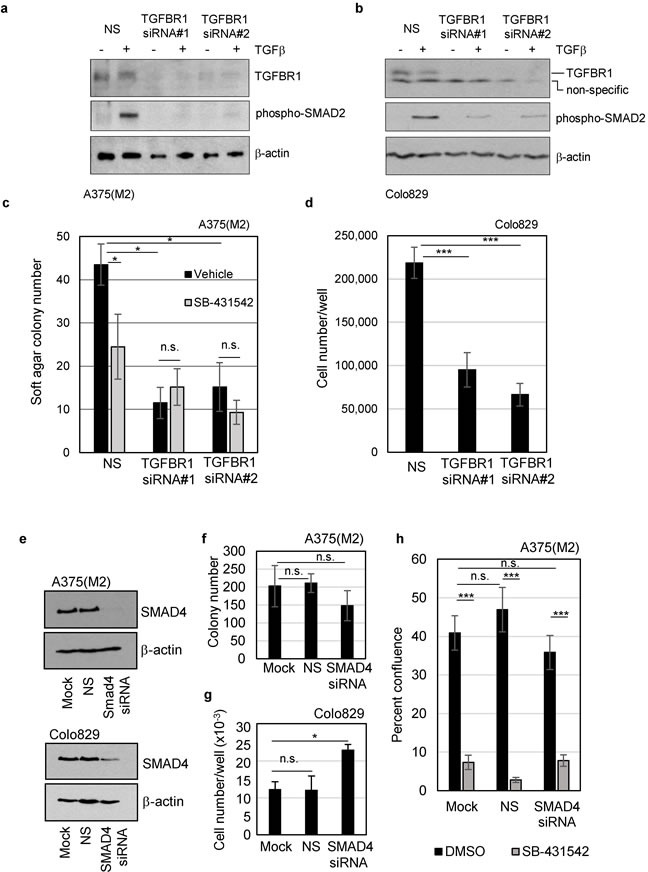
TGFBR1, but not SMAD4, is required for clonogenicity of mutant BRAF^V600E^ melanoma cells A375(M2) **a.**, **c.** and Colo829 **b.**, **d.** cells were transiently transfected with a non-silencing control siRNA (NS) or two independent siRNAs targeting *TGFBR1* (#1 and #2). **a.**, **b.** Western blot analysis of lysates from untreated or TGFβ treated cells (2 hours) were included to confirm a reduction in TGFBR1 expression and decreased signalling *via* phospho-SMAD2 following TGFBR1 knockdown. **c.** A375(M2) knockdown cells were seeded into soft agar assays in the presence of either vehicle control (DMSO, 0.1%) or SB-431542 (10μM). Mean colony numbers (± SD) are given and significant [(*) *p* < 0.05] and non-significant (n.s.) changes in colony number determined by Student's TTEST are indicated. **d.** Colo829 cells transfected for 48 hours with non-silencing, or TGFBR1 siRNA in triplicate were seeded in 6-well plates. Cells were fed by 50% media replacement every 2 days and cell proliferation determined after 11 days. Mean (± SD) cell number is given and analysed for statistical significance by Student's TTEST compared to the non-silencing control. **e.** A375(M2) and Colo829 cells were transiently transfected with a non-silencing control siRNA (NS) or smartpool siRNA targeting SMAD4. Cell lysates were analysed by SDS-PAGE and western blotting for knockdown levels (f, g) Colony formation **f.** or cell proliferation **g.** was determined following SMAD4 knockdown in A375(M2) and Colo829 cells, respectively. Statistical analysis was performed using Students TTEST and n.s indicates non-significant and * indicates *p* < 0.05. **h.** A375(M2) cells were transiently transfected with smartpool siRNA targeting SMAD4, treated with either vehicle control (DMSO, 0.1%) or SB-431542 (10μM) and assayed by live cell imaging for drug sensitivity. Data shown are the means ± SEM percent confluence of 9 wells (4 fields/well) from a representative experiment. Statistical analysis was performed using Students TTEST and n.s indicates non-significant and *** indicates *p* < 0.001.

So far, our data implicate autocrine TGFβ signalling through TGFBR1 as a critical factor in melanoma clonogenicity and tumour formation, however, it was important to rule out off-target effects of the inhibitor. We therefore assessed colony formation following transient transfection with two independent siRNAs targeting TGFBR1. TGFBR1 knockdown (Supplementary Figure 3a) reduced TGFBR1 protein expression and phosphorylation of SMAD2 in response to exogenous TGFβ (Figure 3a and 3b), and recapitulated the effect of chemical inhibition of the receptor. Colony formation and cell proliferation decreased following TGFBR1 knockdown (Figure 3c, 3d, and Supplementary Figure 3b and 3c), confirming that TGFBR1 is required for melanoma colony formation.

To discover whether the canonical SMAD pathway is required for either melanoma cell colony formation or for the inhibitory response to SB-431542, transient knockdown of the co-SMAD, SMAD4 was performed. Knockdown of SMAD4 (Figure 3e) had no significant effect on A375(M2) colony formation (Figure 3f) and significantly increased Colo829 cell proliferation (Figure 3g). These data suggest that SMAD4-dependent signalling is not necessary for colony outgrowth and may, in fact, repress colony growth in Colo829 cells. SMAD4 knockdown also did not block the inhibitory effect of SB-431542 (Figure 3h), indicating that the mechanism of inhibition is SMAD4 independent. In case the levels of knockdown were not sufficient to accurately assess the contribution of SMAD4, we tested a mutant BRAF/SMAD4 null cell line (HT29). These cells were also sensitive to TGFBR1 inhibition (Supplementary Figure 4a and 4b) thus supporting our conclusion that functional SMAD4 is not necessary for inhibition of colony formation by SB-431542.

We next considered any non-canonical signalling pathways that might be affected by TGFβ signalling in melanoma cells. We previously reported that a non-canonical TGFβ/TGFBR1/RHOA signalling pathway is necessary for initiation and maintenance of rodent fibroblast BRAF^V600E^ transformed cultures [31]. Thus, in mutant BRAF human melanoma cells, it seemed plausible that this pathway could be involved in regulating melanoma cell clonogenicity. SB-431542 treatment reduced levels of active-GTP bound RHOA (Supplementary Figure 4c), while transfection of melanoma cells with the exoenzyme C3 transferase to inhibit RHOA [32] mimicked the effect SB-431542 (Supplementary Figure 4d). In addition, overexpression of either constitutively active RHOA, or the constitutively active RHOA specific guanine nucleotide exchange factors Δ558LARG or onco-LBC (to activate endogenous RHOA) [33], blocked the effect of the TGFBR1 inhibitor on colony formation (Supplementary Figure 4e). These data are consistent with our previous findings in rodent fibroblasts.

To gain further insight into the cellular pathways involved in the inhibition of colony formation, we analysed cells by microscopy for division and apoptosis, using BRDU incorporation or fluorogenic apoptosis reagents respectively. Initial experiments revealed that SB-431542 treatment significantly reduced BRDU incorporation but induced little apoptosis (Supplementary Figure 5a and data not shown). We questioned whether an apparently modest reduction in BRDU incorporation was sufficient to account for the dramatic reduction in colony formation and cell proliferation. To investigate in more detail, we generated A375(M2) cell lines stably transfected with an H2B-red fluorescent protein (RFP) fusion protein expression construct to enable kinetic single cell tracking using IncuCyte imaging. Imaging between days four and six of treatment (Supplementary Figure 5b) showed a reduction in their number, and a significant increase in the length of time cells remained in interphase (Supplementary Figure 5c). There were slight increases in the mean number of cells that failed to enter into mitosis or detached upon treatment, but these differences did not reach statistical significance (Supplementary Figure 5d and 5e). TGFBR1 inhibition, therefore, predominantly affects the proportion of cells in S-phase, and significantly affects the clonogenic potential of BRAF mutant cells through effects on the cell cycle.

### BRAF inhibitor resistance

The addiction of mutant BRAF melanoma cells to signalling through TGFBR1 suggests a potential novel therapeutic approach for mutant BRAF-driven cancers. Ideally, not only would a novel treatment be effective as a single agent without evidence of refractory disease (Figure 1), but the novel therapeutic drug would act in combination with existing therapies to enhance their efficacy or prevent the development of resistance. The current therapeutic modality for mutant BRAF metastatic melanoma is treatment with BRAF inhibitors vemurafenib or dabrafenib in combination with MEK inhibitors for suitable patients. However, the development of BRAF inhibitor-resistant disease through a variety of different mechanisms, including the paradoxical activation of the MAPK pathway, remains a significant clinical problem.

**Figure 4:**
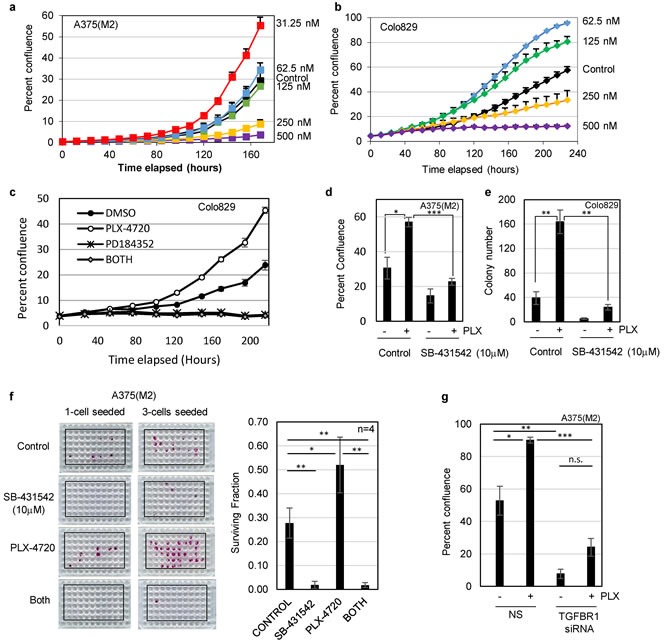
Low dose BRAF inhibitor (PLX-4720) enhances proliferation of drug naïve melanoma cells **a.**, **b.** Cell proliferation assays were carried out by live cell imaging (IncuCyte Zoom). Colo829 (1500 cells/well of 96-well pate) and A375(M2) cells (100 cells/well of 96-well plate) were seeded overnight and treated with PLX-4720 at the concentrations indicated. The mean percent confluence (± SEM) from 8 fields (Colo829) and 9 fields (A375M2) from a representative experiment is shown. **c.** Colo829 cells were treated with solvent control (DMSO, 0.1%), PLX-4720 (62.5nM), the MEK inhibitor PD184352 (2μM) or PLX-4720 + PD184352 (BOTH) and cell proliferation analysed by live cell imaging. The mean percent confluence (± SEM) from 24 fields across 6 wells in a representative experiment is shown. **d.** A375(M2) cells were assayed for cell proliferation for 8 days following treatment with inhibitors of both mutant BRAF (PLX-4720, 31.25 nM) and TGFBR1 (SB-431542, 10μM). Statistical analysis was performed using Students TTEST and * = *p* < 0.05 and *** = *p* < 0.001. **e.** Colo829 cells (40000/10cm dish) were seeded overnight prior to treatment with solvent control (DMSO, 0.1%), PLX-4720 (62.5nM), SB-431542 (10μM) or PLX-4720 + SB-431542. Cells were fed by 50% media replacement every 3 days and colonies were fixed, stained and counted at day 16. Statistical analysis was performed using Students TTEST and * = *p* < 0.05 and ** = *p* < 0.01. **f.** Clonogenicity assays with A375(M2) cells seeded at 1 and 3 cells/well were carried out with vehicle control, SB-431542 (10μM), PLX-4720 (31.25nM) or both drugs (BOTH) for 14 days. Representative plates stained with SRB are shown (left panel). The mean surviving fraction of colonies (± SD) (right panel) was determined (as described in the methods section) from plates seeded with both 1 and 3 cells/well from independent replicate experiments (*n* = 4). Statistical analysis was performed using Students TTEST and * = *p* < 0.05 and ** = *p* < 0.01. **g.** A375(M2) cells were assayed for cell proliferation following transfection with non-silencing (NS) siRNA or siRNA targeting TGFBR1 followed by treatment with PLX-4720 (31.25nM). Statistical analysis was performed using Students TTEST and * = *p* < 0.05, ** = *p* < 0.01, *** = *p* < 0.001 and n.s = not significant.

To assess the potential for a combination therapy targeting both BRAF and TGFBR1, we first tested the sensitivity of previously drug naïve A375(M2) and Colo829 to the mutant BRAF kinase inhibitor PLX-4720 in our clonogenic, low density assay conditions. As expected, at doses exceeding 250nM, growth of both cell lines was inhibited, however, at lower doses, we noted an unexpected significant increase in cell proliferation (Figure 4a, 4b and Supplementary Figure 6a). Suboptimal doses of PLX-4720 induced phosphorylation of ERK (Supplementary Figure 6b), and the enhanced proliferation of Colo829 (Figure 4c) and A375(M2) cells (data not shown) were abrogated by co-treatment with the MEK inhibitor PD184352 (Figure 4c). These data are consistent with low dose PLX-4720 paradoxically activating the RAS-MAPK pathway. Since both cell lines carry BRAF^V600E^ and are wild type for RAS, the most likely interpretation is that low dose PLX-4720 relieves an inhibitory autophosphorylation [34]. Consistent with this hypothesis, low dose PLX-4720 did not promote the proliferation of three melanoma cell lines carrying wild type RAF/RAS (Supplementary Figure 7). Importantly co-treatment of PLX-4720 treated mutant BRAF cells with SB-431542 (10μM) abolished the increase in cell growth caused by low dose PLX-4720 (Figure 4d and 4e). We quantified the effect on clonogenicity [35] and showed that not only did SB-431542 significantly reduce clonogenicity as a single agent, but that the significant increase in clonogenicity induced by PLX-4720 alone was reversed by SB-431542 (Figure 4f and Supplementary Figure 6c). This result was recapitulated by siRNA knockdown of TGFBR1 (Figure 4g).

**Figure 5:**
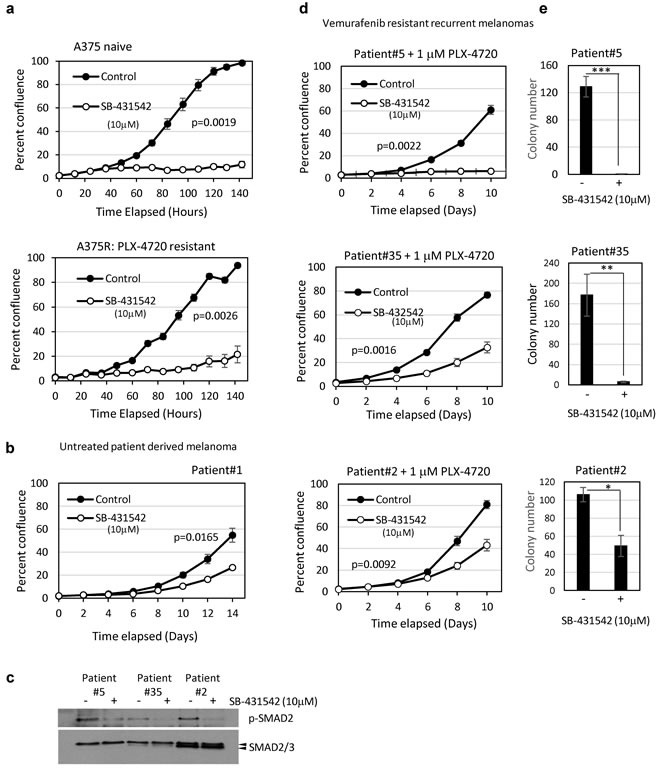
Patient-derived BRAF inhibitor resistant tumour cells are sensitive to TGFBR1 inhibition **a.**, **b.**, **d.** Cells seeded at 500 - 800/well in 96-well plates were assayed for proliferation in the presence of solvent control (DMS0, 0.1%) or SB-431542 (10μM). A375 cells and the PLX-4720 resistant derivative A375R **a.**, patient-derived drug naïve **b.**, and vemurafenib resistant patient tumour derived cell lines **c.**, **d.** were tested. Vemurafenib resistant patient-derived recurrent tumour cells shown in **d.** were routinely cultured in the presence of 1μM PLX-4720. Data is presented as the mean percent confluence (± SEM) from 6 replicate wells, 4 fields/well from representative experiments. Statistical analysis was performed using compareGrowthCurves (Statmod). **c.** Lysates from vemurafenib resistant patient-derived recurrent tumour cells were assayed by western blot for constitutive TGFβ signalling and the response to TGFBR1 inhibition (4 hours, 10μM SB-431542). Phosphorylation of SMAD2 was used as a marker of TGFβ activity. **e.** Vemurafenib resistant recurrent melanoma patient cell lines were seeded in 10cm dishes at 1000 cells (Patient#35, *n* = 4), 16,000 cells (Patient 5, *n* = 4) and 1000 cells (Patient#2, *n* = 3) per dish and treated with solvent control or SB-431542 (10μM). Colonies were stained, counted and the mean colony number ± SD presented. Statistical analysis was carried out by Student TTEST, * = *p* < 0.05, *** = *p* < 0.001.

To investigate further the potential for TGFBR1 inhibitors to prevent vemurafenib resistance, we tested both *in vitro* derived resistant lines (A375R), and patient derived vemurafenib-resistant recurrent tumour cells for sensitivity to SB-431542 (10μM). Patients #2 and #35 (stage IV) achieved a partial response having received vemurafenib for 3 months. Patient #5 (stage IV) had progressive disease and received vemurafenib for 2 months (Supplementary Table 1). The growth of vemurafenib-naïve A375 (Figure 5a) and patient tumour derived cells (Patient#1) (Figure 5b) [18] were both inhibited by SB-431542. Importantly, *in vitro* derived PLX-4720 resistant A375R cells (cultured in the presence of 1μM PLX-4720) were growth inhibited by SB-431542 (Figure 5a). The patient-derived vemurafenib resistant cells had readily detectable levels of phosphorylated SMAD2 that were reduced on SB-431542 treatment, indicating that they all had active autocrine TGFβ signalling (Figure 5c). In addition, all vemurafenib-resistant lines derived from patients were growth inhibited by SB-431542 in proliferation assays (Figure 5d) and in longer term colony formation assays (Figure 5e). Vemurafenib resistant cells therefore retain their sensitivity to inhibitors of the TGFβ signalling pathway.

**Figure 6:**
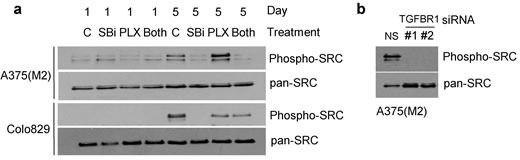
SB-431542 treatment and TGFBR1 knockdown inhibit phosphorylation of SRC **a.** A375(M2) and Colo829 cells were seeded at low density in 10cm dishes in the presence of SB-431542 (SBi) (10μM) and/or PLX-4720 (62.5nM and 31.25nM for A375(M2) and Colo829 cells respectively). Samples treated with both SB-431542 and PLX-4720 are labelled (Both). At Day 1 and Day 5, cells were harvested and analysed by SDS-PAGE and western blotting using the antibodies indicated. **b.** A375(M2) cells were transfected with non-silencing siRNA or siRNAs (#1 and #2) targeting TGFBR1. Cells were seeded at low cell density and after five days harvested for SDS-PAGE analysis and western blotting for the proteins indicated.

Several reports indicate that the development of resistance to BRAF kinase inhibitors may be associated with signalling through SRC-family kinases, and that resistance can be overcome by inhibition of SRC activity [17–19, 36]. We therefore tested whether TGFβ signalling was associated with SRC phosphorylation in A375(M2) and Colo829 cells (Figure 6). We noted that phosphorylated-SRC levels increased during the five day incubation period in cells initially plated at low density. SB-431542 (10μM) (Figure 6a) and TGFBR1 siRNA (Figure 6b) prevented any accumulation of phosphorylated SRC during the time course, and SB-431542 blocked an increase in phospho-SRC levels induced by low-dose PLX-4720 in A375(M2) cells (Figure 6a). An important implication of these data is that inhibition of TGFBR1 signalling may restrict signalling through a known mediator of vemurafenib resistance.

### Zebrafish embryo xenograft metastasis model

**Figure 7:**
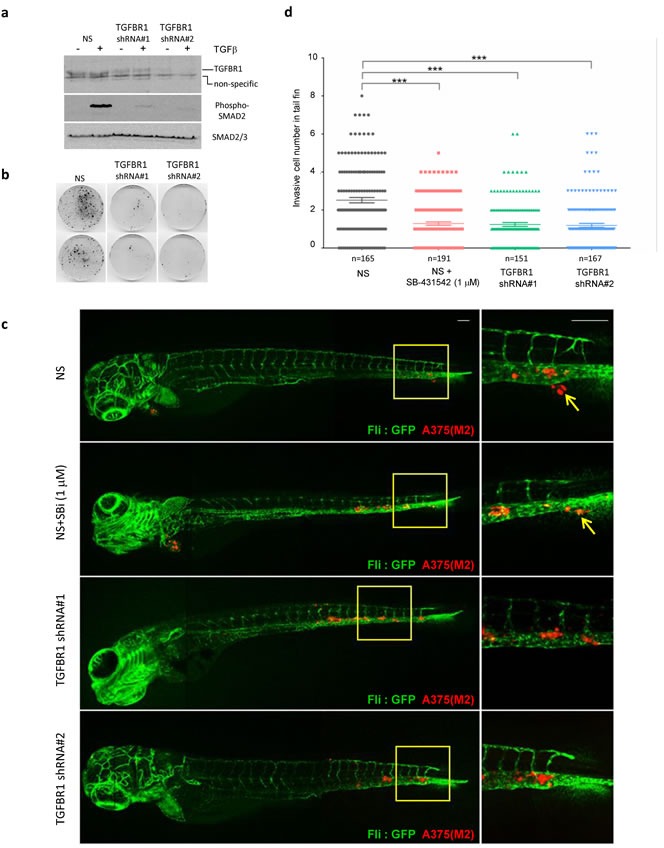
TGFBR1 is required for tumour cell metastasis in xenografted zebrafish **a.**, **b.** A375(M2) cells stably transfected with a non-silencing shRNA control plasmid or two independent shRNA vectors targeting TGFBR1 were assessed by western blotting for a reduction in TGFBR1 expression and signalling in response to exogenous TGFβ addition **a.** and colony formation **b.**. **c.** Cells described in **a.** were labelled with mCherry and implanted into the Duct of Cuvier of zebrafish at 2 days post-fertilization (dpf). SB-431542 (1μM) was added to the egg water of the non-silencing (NS) + SB-431542 group. Confocal images were taken at 4 days post implantation (dpi). Arrows indicate invasive tumour cells, scale bar: 100 μm. **d.** Invasive cell numbers in tail fin of each zebrafish in each group. Statistical analysis was performed using one-way analysis of variance (ANOVA) followed by the Tukey's method for multiple comparison *** *p* < 0.001. Data are combined from four independent experiments and the total number of embryos (n) in each group is indicated.

So far, our murine xenograft assays, and inhibition of clonogenic potential of melanoma cells in low cell density 3D and 2D culture systems, suggest that TGFBR1 inhibitors would be effective in preventing establishment of disease. To further examine whether TGFBR1 inhibitors could effectively treat established cell cultures, we seeded cells at low cell density and progressively delayed addition of drug throughout the lag phase of cell growth. SB-431542 was effective if administered during the lag phase, but delaying treatment until the cells start to exit the lag phase considerably reduced its efficacy (Supplementary Figure 8a). Similarly, seeding cells at higher cell numbers also reduced the efficacy of SB-431542 in both vemurafenib naïve and resistant cells (Supplementary Figure 8b). Given these observations, we posit that cell:cell contact and/or allowing the secretion of growth factors or matrix components has a protective effect against TGFBR1 inhibitors; the implication is that TGFBR1 inhibitors might not be useful as first line, single agents or as debulking therapeutic agents in established solid tumours. Nevertheless, the dependence of melanoma cells on TGFBR1 for clonogenicity suggests that TGFBR1 inhibitors could be effective in preventing spread or outgrowth of micrometastases. To test this hypothesis we used a zebrafish embryo metastasis model [37, 38] to visualise and quantify numbers of invasive melanoma cells. This model has been used successfully to examine the effect of SB-431542 on breast cancer cell invasion [39]. We generated stable TGFBR1 knockdown A375(M2) cell lines using LMP-TGFBR1 shRNA plasmids, which had reduced TGFBR1 protein expression and reduced capacity to signal (Figure 7a) as well as reduced ability to form colonies on plastic (Figure 7b). The stable lines were labelled with the mCherry fluorophore, injected into the Duct of Cuvier (DoC) of zebrafish embryos and the numbers of invasive cells in the avascular tail fins were analysed. Representative con-focal images of metastatic spread into the tail fin are shown in Figure 7c. Control non-silencing (NS) A375(M2) cells were capable of metastatic spread into the fish fin (arrows indicate micro-metastases). Using doses of 1μM SB-431542 (SBi), we observed a significant decrease in the ability of SB-431542 treated NS cells to invade. Stable TGFBR1 knockdown also reduced colonisation of zebrafish tissue (Figure 7d). Our data overall suggest that TGFBR1 inhibitors would reduce the metastatic burden in BRAF mutant melanoma by preventing invasion and/or outgrowth of metastatic colonies.

## DISCUSSION

The outcome for patients with advanced melanoma has improved dramatically in recent years. The development of targeted therapy of the MAPK pathway, and advances in immunotherapy have resulted in improvements in median survival from 9 months to 25-31 months. However, long-term prognosis remains uncertain. For targeted therapy using BRAFi, drug resistance mechanisms identified to date are numerous, and there is no established effective second-line targeted therapy for patients progressing on combination BRAFi + MEKi. Often, drug-resistance mechanisms involve induction of either an autocrine, or a paracrine, drug-induced secretome which helps to promote expansion and dissemination of the drug-resistant cells [15] and/or protect potentially sensitive tumour cells from the inhibitory effects of the chemotherapeutic agent [15, 19, 21]. Phosphorylated ERK and SRC are frequently elevated in resistant tumours [18] suggesting that paradoxical activation of the MAPK pathway and growth factor signalling are involved in resistance development. In this study we investigated the potential of targeting TGFβ1 as second-line therapy for advanced melanoma.

We found that autocrine TGFβ signalling through TGFBR1 is an intrinsic requirement for the clonogenic potential of mutant BRAF transformed cells, indicating that mutation of BRAF may be useful as a biomarker for TGFβ tumour promoting activity in melanoma. Although TGFβ levels are elevated post-vemurafenib treatment [20, 21], our data suggest that a TGFβ/TGFBR1-dependent state is an adaptation to the presence of mutant BRAF. Thus, with pro-tumourigenic autocrine TGFβ signalling pathways having already been established, elevated levels of signalling during therapy are perhaps more readily selected for than would otherwise be the case. How the initial switch from tumour suppressor to tumour promoter function of TGFβ in melanocytes is mediated by mutant BRAF remains to be determined.

Our *in vitro* assays were specifically designed to mimic conditions of cellular stress (i.e. low density 2D and anchorage independent colony formation assays) to more accurately assess the clonogenic potential of melanoma cells and their cancer stem-cell like properties. TGFBR1 inhibition was highly effective in inhibiting growth of both naïve and vemurafenib-resistant cells when administered during the lag phase of growth; less so when cell seeding numbers were increased. The implication of these data is that targeting TGFβ/TGFBR1 may not be an effective therapeutic strategy in established tumours. A secondary consideration is that TGFBR1 inhibition affected proliferation of the tumour cells without inducing apoptosis, and so may not result in significant tumour shrinkage. As a consequence, we predicted that inhibiting TGFβ/TGFBR1 signalling would more likely be effective in preventing tumour metastasis and outgrowth of micrometastasis, rather than reducing established tumour burden. Indeed, our murine and zebrafish xenograft models show that targeting autocrine TGFβ secretion and TGFBR1 kinase activity inhibits xenograft tumour establishment in mice and prevents metastatic spread in zebrafish tissues. TGFBR1 inhibitors therefore may have potential as an adjuvant therapy in high risk, resected disease, or a maintenance therapy in patients responding to BRAF inhibitors. The inhibition of glioblastoma cancer initiating (stem) cells by TGFBR1 inhibitors [40, 41] is consistent with our data, and provides support for our conclusion that TGFBR1 activity is required for melanoma stem-cell like properties.

Given the vast number of context specific genes regulated by TGFβ, it is likely that a number of different downstream effectors will mediate the autocrine TGFβ-induced promotion of melanoma cell growth and drug-resistance. We showed that although melanoma cells rely on TGFBR1 kinase activity, they do not require SMAD4 for either colony formation, or for the response to TGFBR1 inhibition. Signalling *via* RHOA, however, rescued the effect of SB-431542 which is both consistent with our previous analysis of rodent fibroblast transformation [31], and with a role for TGFβ-activated non-canonical signalling pathways in this response. The establishment of an adaptive autocrine TGFβ/TGFBR1 signalling pathway through RHOA following BRAF mutation may be necessary to overcome CDKN1A expression and growth arrest induced as a response to oncogenic stress [31, 42]. Consistent with this are our data showing CDKN1A induction by disrupting TGFβ signalling both *in vitro* and *in vivo* (Figure 2 and Supplementary Figure S2). Interestingly, inhibition of ROCK (using Y27632), a downstream target of RHOA signalling, also induces CDKN1A in melanoma [43], and ROCK1 has been identified as a potential candidate for combinatorial therapy with BRAF inhibitors. In these studies inhibition of ROCK1 sensitises melanoma cells to PLX-4720 [44]. Our data now suggest that the involvement of ROCK1 in resistance mechanisms is potentially a result of TGFβ/TGFBR1/RHOA signalling. Although the SMAD-dependent induction of RHO GEFs has been described in other studies, [45, 46] how TGFBR1 directly activates RHOA in melanoma cells in a SMAD-independent manner is unclear at present.

Several other TGFβ target genes have been implicated in melanoma biological responses. Melanoma cells exposed to the high levels of exogenous TGFβ present in bone, upregulate osteolytic genes (including *IL-11*, *PTHrP*, and *CTGF*) which may aid more effective colonisation of this metastatic niche. Blocking receptor function by over-expression of the natural inhibitor SMAD7 extended survival of mice xenografted with SMAD7 expressing melanoma cells. However, a causal role for the TGFβ-regulated osteolytic genes in bone metastasis was not directly demonstrated [26]. Similarly, the balance between the TGFβ target gene *GLI2* and the melanocyte specific isoform of *MITF* (*M-MITF)* appears important for invasion through matrigel *in vitro*, with high *GLI-2/*low *M-MITF* correlating with invasion. However, these expression profiles were independent of BRAF mutation status and did not correlate with either proliferation *in vitro* or with subcutaneous xenograft tumour establishment [47]. We suspect that TGFβ target genes induced by exogenous TGFβ exposure may be quite different from those genes regulated by non-canonical signalling as a result of autocrine TGFβ ‘addiction’ established following BRAF mutation. Further work to identify which are the key TGFβ target genes involved in both promoting these stem-cell properties, and in driving drug-resistance, is underway and we expect that these studies will suggest novel, selective therapeutic targets.

We show that drug-naïve melanoma cells are growth promoted by low-dose PLX-4720, likely by paradoxical activation of the MAPK pathway. This may have important implications clinically, since low doses of bioavailable BRAFi reaching some tumour tissue could actually potentiate tumour growth. Importantly, we show that both paradoxically activated, previously drug naïve cells, as well as vemurafenib resistant cells, retain sensitivity to TGFBR1 inhibitors. In addition, SB-431542 prevented phosphorylation of SRC which is frequently associated with vemurafenib resistance, suggesting that TGFBR1 inhibitors would prevent relapse with vemurafenib-resistant metastases. How SB-431542 regulates SRC activation is currently under investigation in our laboratory. It will be important to test the sensitivity of BRAFi/MEKi resistant cells derived from patients treated with combination therapy when established. Nevertheless, we predict that targeting an independent signalling pathway may have some advantages over combination therapies which target different components of the same signalling pathway. In addition, blocking the immunosuppressive effects of TGFβ could potentiate the efficacy of immune based therapeutics. Since dependence on TGFβ signalling appears to be universal in mutant BRAF melanoma cells, targeting TGFβ or downstream effectors may also provide useful therapeutic options for blocking metastatic outgrowth of vemurafenib refractory disease which occurs in approximately 20% of patients receiving treatment. There are currently a number of TGFβ pathway inhibitors progressing through Phase 1-3 clinical trials [48]. The small molecule TGFBR1 inhibitor Galunisertib is being evaluated in cancer patients with unmet need. This inhibitor is deemed tolerable, with an acceptable margin of safety when administered using intermittent dosing regimens [49], demonstrating that TGFBR1 inhibitors are suitable for clinical use and may provide new opportunities for therapy of BRAF-inhibitor resistant cancer.

## MATERIALS AND METHODS

### Western blotting

Cell lysates were analysed by SDS-PAGE using the following antibodies: PO_4_-SMAD2 (Ser465/467) (rabbit polyclonal, #3101, Cell Signalling Technology [CST]), SMAD2 (mouse monoclonal, C16D3, CST), SMAD2/3 (mouse monoclonal, Clone 18, BD transduction Laboratories), SMAD4 (mouse monoclonal, B-8, Santa Cruz Biotechnology), TGFBR1 (rabbit polyclonal, V-22, Santa Cruz Biotechnology), CDKN1A (rabbit polyclonal, C19, Santa Cruz Biotechnology), RHOA (mouse monoclonal, 26C4, Santa Cruz Biotechnology), PO_4_-SRC (Tyr416) (rabbit monoclonal, D49G4, CST), SRC (rabbit monoclonal, 36D10, CST), PO_4_-p44/p42 MAPK (ERK1/2) (Thr202/Tyr404) (rabbit polyclonal, #9101, CST), p44/p42 MAPK (ERK1/2) (rabbit polyclonal, #9102, CST), β-actin (mouse monoclonal, AC-74, Sigma). Secondary HRP-conjugated antibodies (Dako) and enhanced chemiluminescence (GE Healthcare) was used to detect bound antibody.

### Cell culture

Details of the cell lines and media supplements used are shown in Supplementary Table S1. All cells lines were tested regularly for mycoplasma contamination by the Institute's mycoplasma testing service. Patient derived cell lines were passaged for approximately 1 month. Where indicated the cells were transfected with Lipofectamine or Lipofectamine 2000 (Invitrogen) using the following plasmids; pRK5 C3-transferase and pEF-Flag LARG Δ558 (kind gifts of R. Grosse), pRK5-RhoA V14 (kindly supplied by Alan Hall), pSR-Flag onco LBC (kindly supplied by Mike Olson), or pSuper-TGFβ1. LMP-scrambled non-silencing (NS) and LMP-TGFBR1 shRNA constructs were generated in house with the following hairpin sequences:

NS - 5′ CGAGAAGGTATATTGCTGTTGACAGTGAGCGACT CATAGCGATGTGAACTCAATAGTGAAGCCACAGA TGTATTGAGTTCACATCGCTATGAGCTGCCTACTG CCTCGG -3′;

TGFBR1#1 - 5′ TCGAGAAGGTA TATTGCTGTTGACAGTGAGCGACTCATAGAGAT TTGAAATCAATAGTGAAGCCACAGATGTATTGAT TTCAAATCTCTATGAGCTGCCTACTGCCTCGG -3′;

TGFBR1#2 - 5′ TCGAGAAGG TATATTGCTGTTGACAGTGAGCGACAGTGTAATA AAGTCAATTAATAGTGAAGCCACAGATGTATTAAT TGACTTTATTACACTGCTGCCTACTGCCTCGG. -3′.

Cells were transfected with either Oligofectamine or HiPerFect (Qiagen) to introduce, at a final concentration of 20 - -50nM, the following siRNA; allstars negative control, TGFBR1 [HS_TGFBR1_6 (TGFBR1#1) and HS_TGFBR1_7 (TGFBR1#2) (Qiagen)] or SMAD4 (Dharmacon smartpool). Mock transfections (no siRNA) were included in each experiment. A375(M2) pSuper or pSuper-TGFβ1 stable cell lines were selected and maintained in 0.6mg/mL puromycin. A375(M2) histone H2B-RFP stable cell lines were selected and maintained in 800μg/mL G418, and LMP-scrambled or LMP-TGFBR1 shRNA derivatives were maintained in 800μg/mL G418 plus 1μg/mL puromycin. Where indicated the cells were treated with SB-431542 (Tocris) [50], PLX-4720 (Selleck Chemicals) or PD184352 (Cell Signalling) (prepared in DMSO).

### Soft agar assay

Soft agar assays were carried out essentially as previously described [31]. Briefly, six well plates were coated in 2mLs of media supplemented with 0.9% low melting point agar (Invitrogen). 2mL cells (1x10^4^/mL) in media supplemented with 0.45% low melting point agar were overlaid with either SB-431542 or vehicle control. Wells were fed twice weekly for 2-4 weeks, and the number of colonies (> 80μm in diameter) in nine fields of view was scored using an Olympus CKX41 microscope, fitted with a 4X objective and an eyepiece graticule (250μm gradations). Statistical analyses were carried out by Students TTEST unless stated otherwise.

### Proliferation assay

Cell proliferation kinetics were either monitored using an IncuCyte Zoom^TM^ imaging system and software (percent confluence) (Essen Biosciences), or by trypsinisation and cell counts using a Casy cell-counter (model TT, Innovatis).

### Colony formation and clonogenicity assays

Colony formation: Cells were seeded in 10cm dishes at an appropriate density to form approximately 250 discrete colonies after 2-3 weeks in culture. Colonies were fixed in methanol and stained with toluidine blue/borax solution for counting.

Clonogenicity: Cells were seeded overnight at 1 and 3 cells/well in 60 wells of a 96-well plate, prior to treatment. Wells were fed twice weekly, and wells examined by light microscopy. After approximately two weeks, media was removed, colonies fixed in methanol and stained with 0.4% (w/v) sulforhodamineB (SRB)/1% acetic acid. Colonies > 50 cells in size were counted, and the plating efficiency and surviving fractions after drug treatment determined according to Franken et al [35].

### TGFβ1 ELISA

The TGFβ1 assay has been described previously [31]. Briefly, cells were cultured in media containing 0.1% FBS for 24 hours. Media was harvested and the cells trypsinized and counted. The media was acid treated to activate latent TGFβ, and TGFβ1 levels determined by ELISA using anti-TGFβ1 (MAB1835) (capture antibody) and biotinylated anti-TGFβ1 (BAF240) (detection antibody). Recombinant hTGFβ1 (Peprotech) was used as a standard. Results were expressed as TGFβ1 produced per 1x10^5^ cells/hour.

### Mouse xenografts

Nude mouse subcutaneous xenograft experiments were performed according to Home Office guidelines and were approved by the local research and ethics committee (BICRLREC). 1x10^6^ cells were injected subcutaneously into the flank of CD1 nude mice (*n* = 8) (Charles Rivers). Palpable tumours were observed 8 days post-injection and tumour volumes were calculated using calliper measurement and the formula V = (E^2^xA)/2 where E = shortest and A = the longest diameter measurement.

### Immunohistochemistry

Sections from formalin, paraffin embedded, pSuper (Control shRNA) or pSuper-TGFβ1 shRNA tumours were stained for CDKN1A (M19, Santa Cruz Biotechnology) using an Envision kit (Dako) according to the manufacturer's instructions. The sections were counter stained with Haemotoxylin and were scored for CDKN1A expression. One representative field of view (that contained a minimum of 350 cells) was scored (blind) for each tumour.

### IncuCyte zoom and analysis

An IncuCyte Zoom live cell imaging microscope (Essen Biosciences) with 10x objective and data management software was used to monitor kinetic cell proliferation. The mean ± SEM percent confluence from four phase-contrast images/well, with a minimum of 3 replicate wells/treatment was determined according to software processing definitions as recommended by the manufacturer. Statistical analysis was carried out using Graphpad software and pairwise comparisons using the compareGrowthCurves function (statmod, R project, 10,000 permutations).

### Embryo preparation and tumor cell implantation

Zebrafish and embryos were raised, staged and maintained according to standard procedures. The Institutional Committee for Animal Welfare of the Leiden University Medical Center (LUMC) approved this study. Tg(Fli1:GFP) zebrafish embryos were dechorionated at two days post-fertilisation (dpf). Single cell suspensions of melanoma cells were prepared in PBS and kept at 4°C before implantation. The cell suspension was loaded into borosilicate glass capillary needles (1 mm O.D. × 0.78 mm I.D.; Harvard Apparatus) and the injections were performed using a Pneumatic Picopump and a manipulator (World Precision Instruments, Stevenage, UK). Dechorionated embryos were anaesthetised with 0.003% 3-amino benzoic acid ethyl estertricaine, (Sigma)] and mounted on 10 cm Petridishes coated with 1% agarose. Approximately 200 cells were injected at the duct of Cuvier (DoC). Implanted zebrafish embryos were maintained at 33°C. Zebrafish in the Non-Silencing (NS) + SB431542 group were treated with 1μM SB-431542 added to the eggwater. All implantations were repeated at least three times with at least 30 embryos per group.

### Microscopy and analysis of zebrafish

Zebrafish embryos were fixed with 4% paraformaldehyde for two hours at room temperature. Embryos were imaged in PBS/0.1% Tween-20 (Merck, Amsterdam, Netherlands) using a Leica SP5 STED confocal microscope (Leica, Rijswijk, Netherlands). Confocal stacks were processed for maximum intensity projections with Image J. Images were adjusted for brightness and contrast, and overlays created using Adobe Photoshop CS6. Statistical analysis was performed using Prism 4 software (GraphPad, La Jolla, USA). Results are expressed as the mean ± SD. One-way analysis of variance (ANOVA) were performed followed by the Tukey's method for multiple comparison. *P* < 0.05 was considered to be statistically significant (*0.01 < *P* < 0.05; **0.001 < *P* < 0.01; *** *P* < 0.001). In one experiment the results were scored blinded; all results were confirmed by an independent observer.

## SUPPLEMENTARY VIDEOS




