# Supplementary material for: Mutational activation of BRAF confers sensitivity to transforming growth factor beta inhibitors in human cancer cells
Source: Oncotarget. 2016 Nov 9;7(50):81995–2012. doi: 10.18632/oncotarget.13226 (PMC5347669; doi:10.18632/oncotarget.13226)
Supplement: Supplementary file 2 [file oncotarget-07-81995-s002.pdf]

Mutant BRAF confers transforming growth factor β addiction

Supplementary materials and methods

Establishment of a NIH3T3-CAGA$_{12}$ bioassay

NIH3T3 cells were co-transfected at a 10:1 ratio with pGL3-CAGA$_{12}$-luciferase reporter construct (Dennler et al. 1998) and pTK-neo to confer G418 resistance. Stable cell clones were maintained in 400μg/mL G418, and tested for luciferase induction over a range of TGFβ1 concentrations (Supplementary Figure S1a). Luciferase activity was determined using the Luciferase Assay System (Promega) as recommended by the manufacturer and is expressed as the mean relative light units (RLU) ± s.d. of a minimum of triplicate wells. The stable cell clone activated by the widest linear range of TGFβ concentrations was selected for the bioassay. Acidification of test media was carried out prior to the assay to activate latent TGFβ, briefly, media was acidified by addition of 20μL 1N HCL/100μL sample, mixed and incubated for 10 mins at room temperature. The acid was neutralised by addition of 13μL 1.2 N NaOH/0.5M HEPES, media mixed and assayed immediately. Assays +/- activation (with the activated value multiplied by 1.3 to correct for dilution) were compared. Samples were diluted if required to ensure values were within the linear range of the assay – typical dilutions are between neat and 1:6.

BRDU labelling

A375(M2) cells were seeded at 1000 cells/well in 24 well plates with and without SB-431542 (10μM). After 6 days cells were labelled with 30μM BrdUrd (Sigma) for 30 minutes at 37°C. Cells were trypsinised, pooled and fixed in 80% ethanol. Cells were resuspended in prewarmed 1mg/mL pepsin in 30mM HCL and incubated for 30 minutes at 37°C. After centrifugation, cells were resuspended in 2M HCL for 15–20 minutes and then washed. Cells were then stained with mouse anti-BRDU

(Beckton Dickinson) followed by FITC-conjugated goat anti-mouse secondary antibody (Dako). Cells were then either stained with propidium iodide for analysis by flow cytometry, or cytospun for analysis by immunofluorescent microscopy.

RhoA activation assay.

Levels of active, GTP-bound RHO A were determined as previously described (Fleming et al). Briefly, cells were grown in low serum (0.1%) media for 18 hours and then incubated with SB-431542 or vehicle control. After 15 minutes the cells were lysed in 50mM Tris pH 7.2 at 4°C, 150mM NaCl, 1% TX-100, 10mM MgCl2 supplemented with 1x complete anti-protease inhibitors (Roche), clarified at 16,000*g* for 2 minutes at 4°C and mixed with 70µg of Rhotekin Rho binding domain (RBD) beads for 45 minutes at 4°C. The beads were washed in lysis buffer and eluted in SDS-PAGE loading buffer. GST-Rhotekin RBD beads were purified exactly as described by Nimnual et al. The GTP-bound RHOA, and input lysates were analysed by SDS-PAGE.

References

Dennler S, Itoh S, Vivien D, ten Dijke P, Huet S, Gauthier JM. Direct binding of Smad3 and Smad4 to critical TGF beta-inducible elements in the promoter of human plasminogen activator inhibitor-type 1 gene. EMBO J. 1998 Jun 1;17(11):3091-100.

Fleming YM, Ferguson GJ, Spender LC, Larsson J, Karlsson S, Ozanne BW, et al. TGF-beta-mediated activation of RhoA signalling is required for efficient (V12)HaRas and (V600E)BRAF transformation. Oncogene. 2009;28(7):983-93. Epub 2008/12/17. doi: 10.1038/onc.2008.449. PubMed PMID: 19079344.

Nimnual AS, Taylor LJ, Bar-Sagi D. Redox-dependent downregulation of Rho by Rac. Nat Cell Biol. 2003;5(3):236-41. doi: 10.1038/ncb938. PubMed PMID: 12598902.

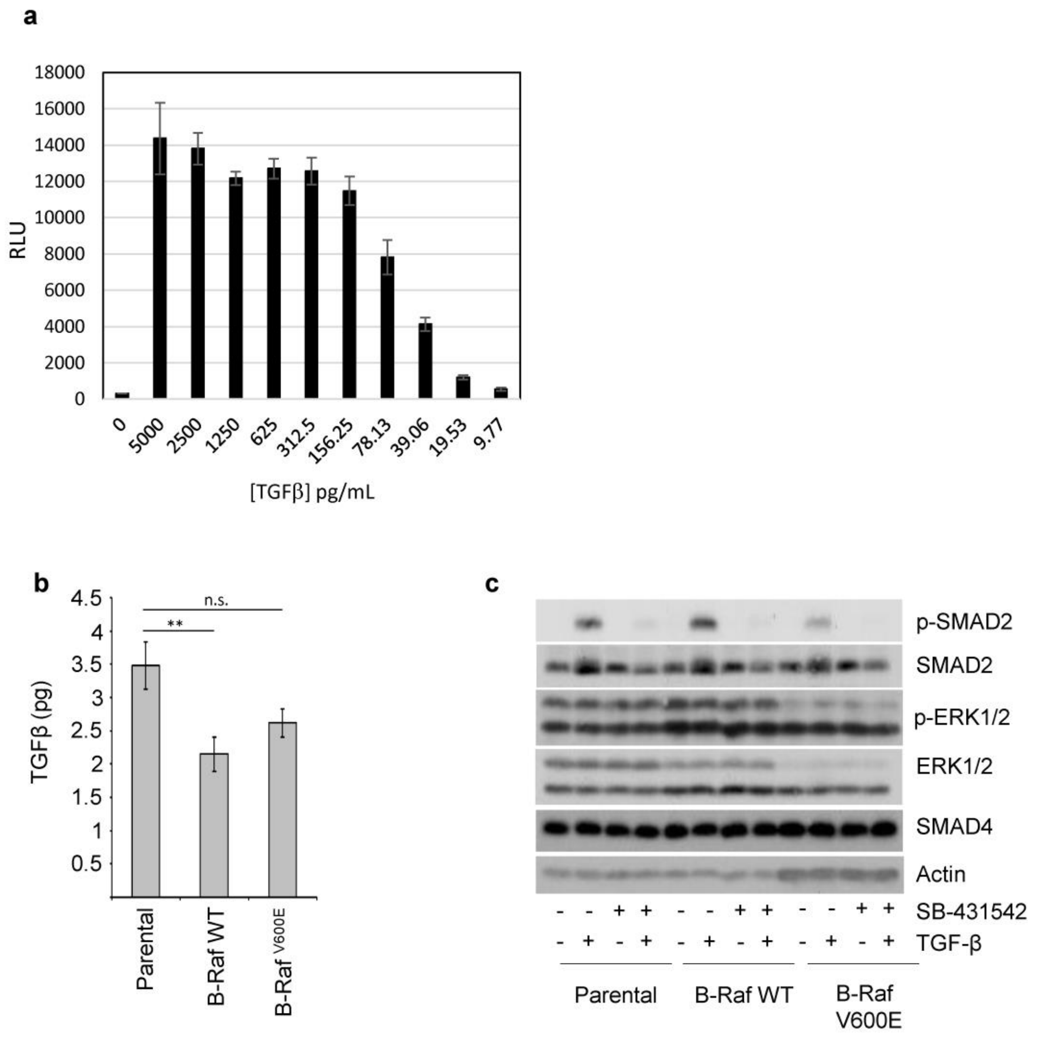

Supplementary Figure 1. (a) NIH3T3 pGL3-CAGA$_{12}$-luciferase stable cell clones were tested for sensitivity to exogenous TGFβ1 addition. Relative light units (RLU) were determined using the Luciferase Assay System kit (Promega) and are expressed as the mean ± s.d of six replicate wells. **(b)** TGFβ levels were analysed using the NIH3T3 CAGA$_{12}$ luciferase bioassay and are expressed as the amount (pg) of TGFβ1 produced by 1x10^5 cells/hour. **(c)** Melan-a cells described in Figure 1A were incubated with 10µM SB-431542 or vehicle control before being treated with 40 pM TGFβ for 1 hour. Protein was extracted and analysed by SDS-PAGE and western blotting for the proteins indicated. A western blot for Actin was included as a loading control.

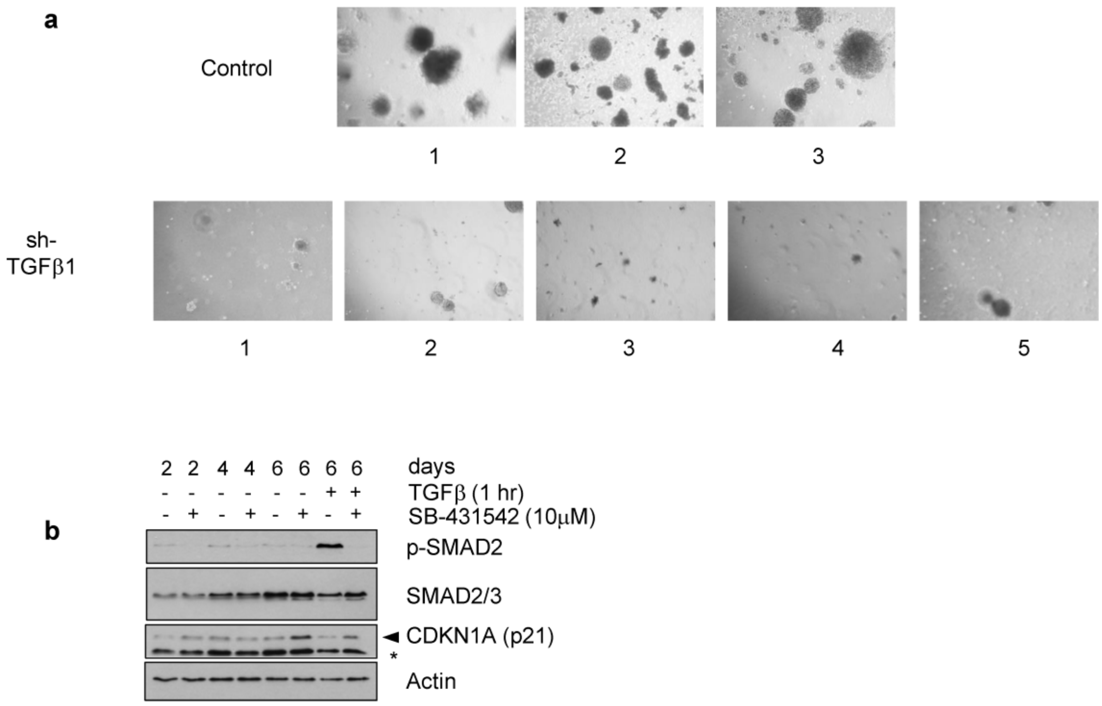

Supplementary Figure 2. Colony formation requires the autocrine production of TGFβ1. (a) A375(M2) clones stably expressing a control shRNA, or an shRNA targeting TGFβ1 (sh-TGFβ1) were seeded into a soft agar assays and incubated for 4 weeks prior to colony counting by light microscopy. **(b)** A375(M2) cells were incubated with 10μM SB-431542 or vehicle control for the times indicated. At six days, duplicate samples were either left untreated or treated with 40 pM TGFβ for 1 hour. Protein was extracted and analysed by SDS-PAGE using antibodies specific for the proteins indicated. The asterisk denotes a non-specific band.

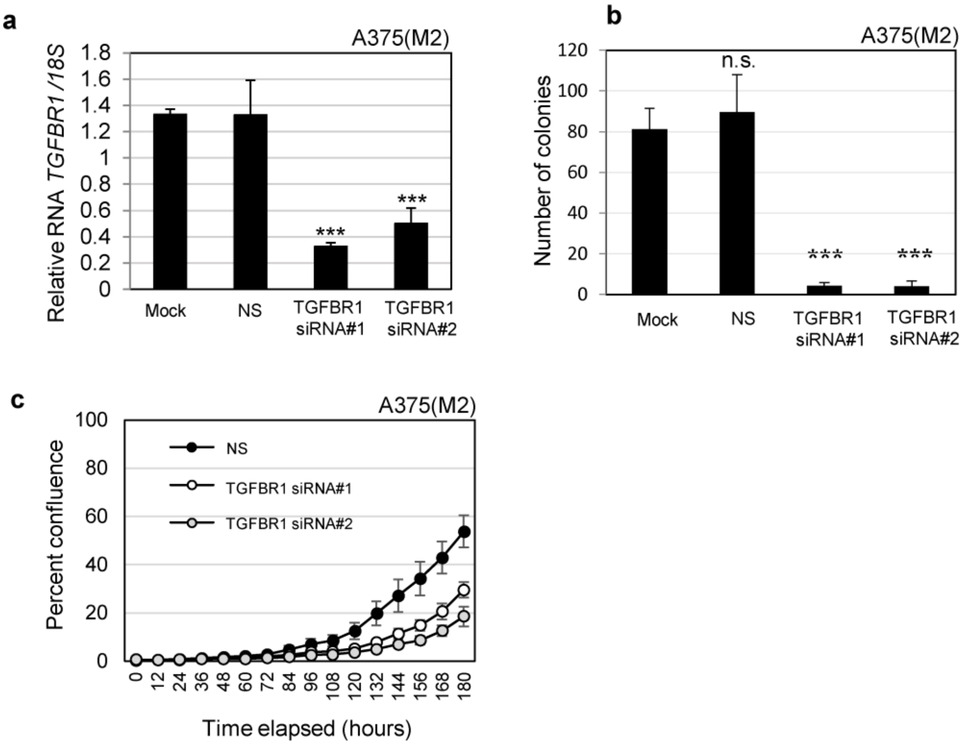

Supplementary Figure 3. TGFBR1 knockdown inhibits the clonogenicity of mutant BRAF^{V600E} melanoma cell lines. (a-c) A375(M2) cells were transiently transfected with a non-silencing control siRNA (NS) or two independent siRNAs targeting TGFBR1. **(a)** qPCR analysis of TGFBR1 RNA expression levels (normalised to 18S) following TGFBR1 knockdown. **(b)** 14 day colony formation assay on plastic of A375(M2) TGFBR1 knockdown cells described in (a). Statistical analysis was performed by Students TTEST in comparison with the mock control. Non-significant (n.s.), p<0.001 (***) **(c)** Growth kinetics of A375(M2) TGFBR1 knockdown cells measured by live-cell imaging (IncuCyte Zoom).

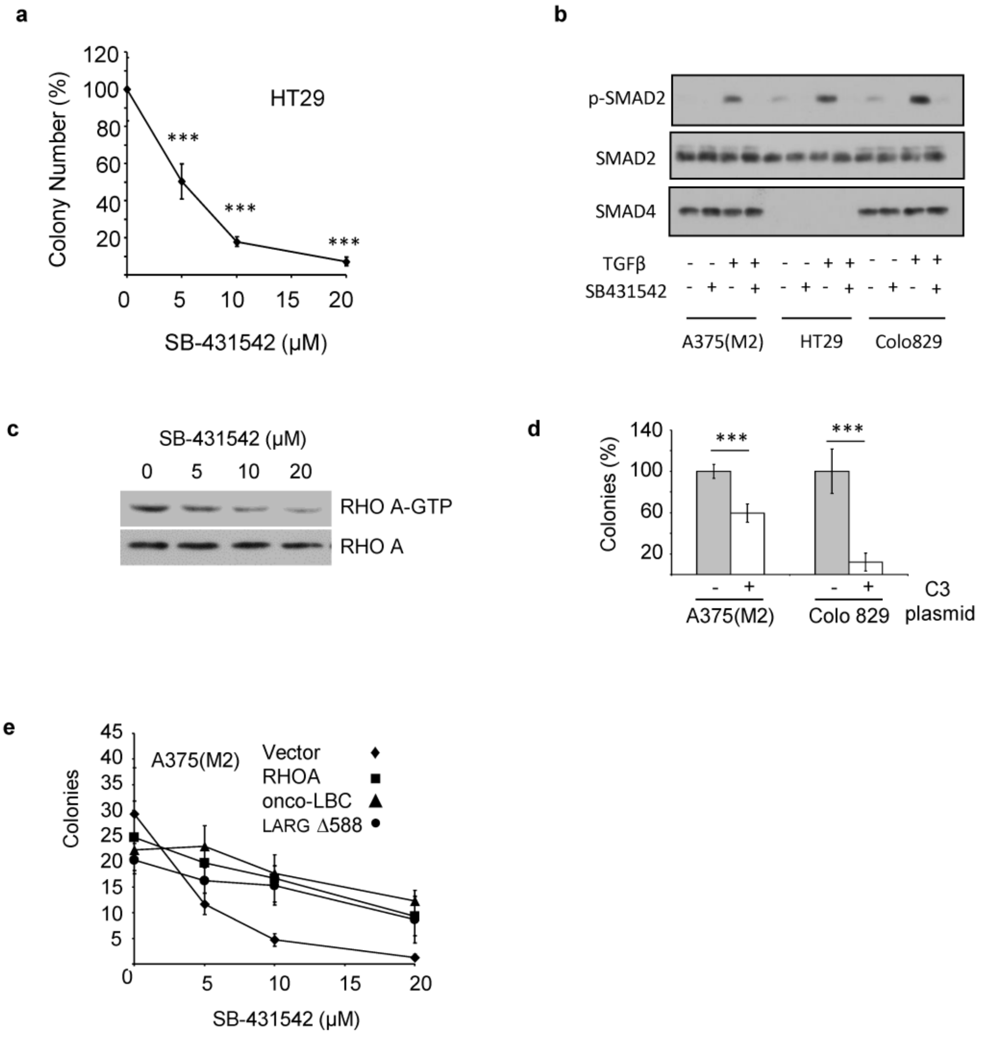

Supplementary Figure 4. Unlike TGFβR1, SMAD4 is not required for sensitivity to SB-431542 or for the clonogenicity of mutant $BRAF^{V600E}$ cell lines. (a) HT29 SMAD4 null cells were seeded in soft agar in the presence of the indicated concentration of SB-431542 or vehicle control (0.2% DMSO). Colonies were counted and presented as the mean ± SD colony number as a percentage of the vehicle control. n=6 from 2 independent experiments. **(b)** Cells were incubated with 10μM SB-431542 or vehicle control before being treated with 40 pM TGF-β for 1 hour. Protein was extracted and analysed by SDS-PAGE using PO_4-Smad2, Smad2 or Smad4 antibodies. **(c)** Colo829 cells were treated with the indicated concentration of SB-431542 for 15 minutes before being analysed for the levels of active RHO A-GTP or RHO A using the GST Rhotekin-RBD pull down assay and western blotting. **(d, e)** Cells were transfected with the listed expression constructs and seeded into soft agar with the indicated concentration of SB-431542. Colonies were counted and presented as the mean ± SD colony number. Statistical analysis was performed using Student’s TTEST.

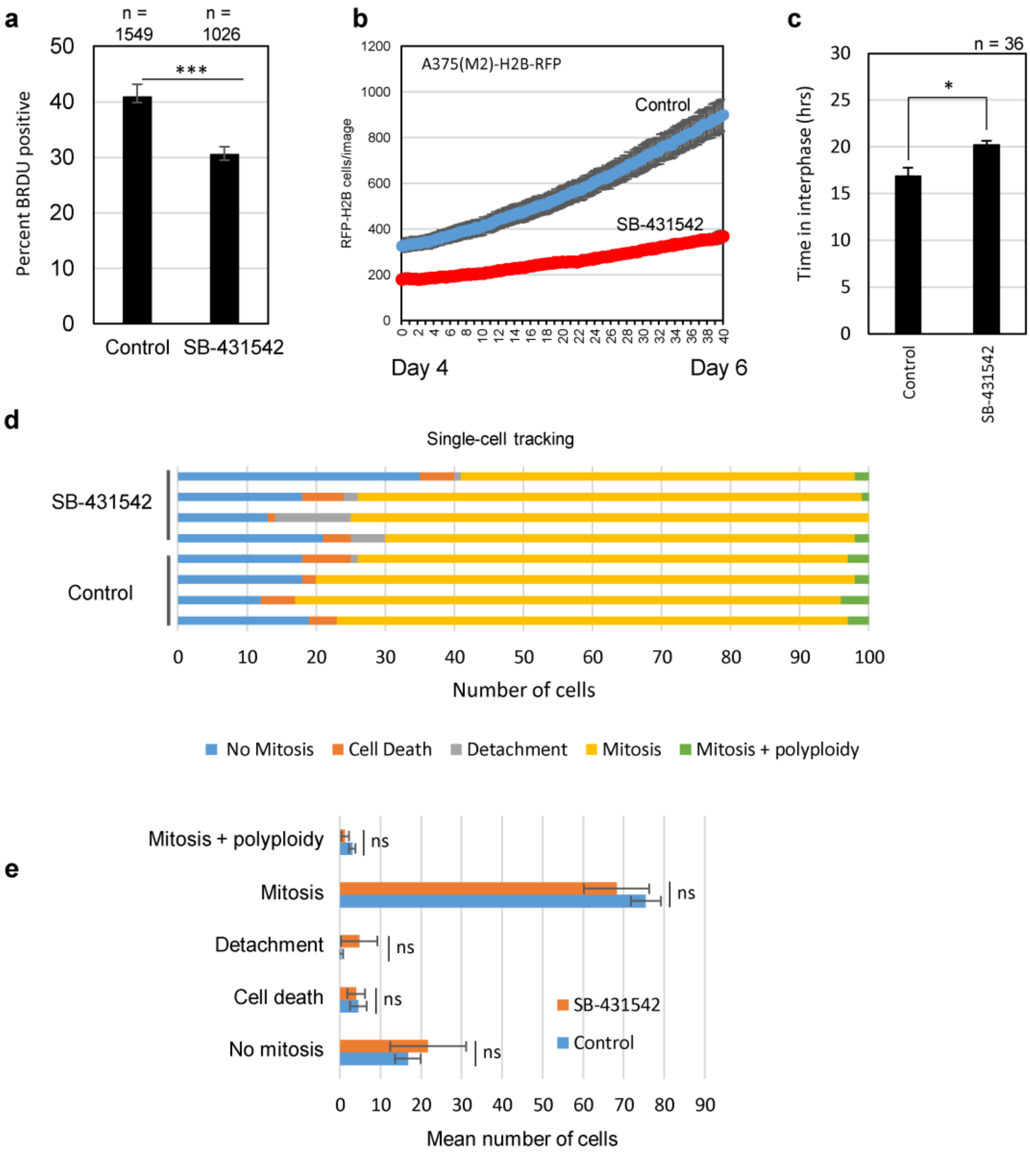

Supplementary Figure 5. Analysis of the proliferation defect in melanoma cells following TGFBR1 inhibition (a) A375(M2) cells were treated with solvent control (DMSO, 0.1%) or SB-431542 (10μM) for 6 days, labelled with BRDU-FITC and analysed by light microscopy (n= number of cells analysed). **(b)** A375(M2) cells were stably transfected with an H2B-RFP expression plasmid and treated with either solvent control (Control) or SB-431542 (10μM). Cells were imaged using phase contrast and red fluorescence every 2 hours and movies generated between days 4 and 6 post-treatment. The mean (± SEM) number of RFP positive cells per field under Control (blue line) and SB-431542 treated (red line) conditions is indicated. **(c, d)** Live cell imaging analysis of movies described in (b). Single RFP positive cells were tracked, and the time in interphase (c), or cell fates as listed in (d) were recorded (n=100 cells in four fields): cells not entering into mitosis (no mitosis), cells rounding up and fragmenting (cell death), cells detaching and leaving the field of view (detachment), cells completing mitosis (mitosis) and cells entering mitosis but failing to undergo cytokinesis (mitosis + polyploidy). (**e**) Bar chart depicting the mean number of cells in each category from replicates shown in (d). ns= not significant by Student's TTEST.

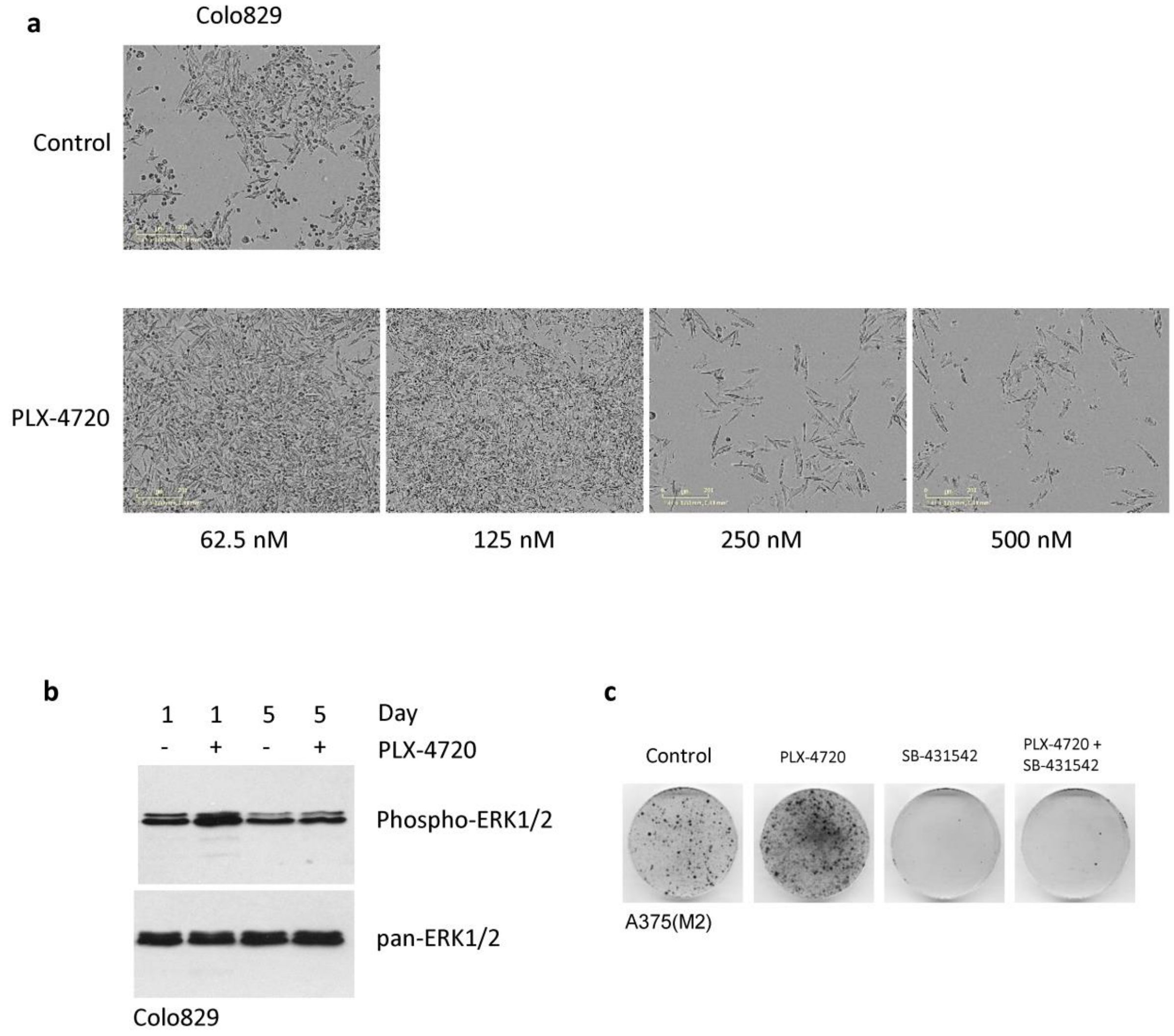

Supplementary Figure 6. PLX-4720 naïve wild type RAS/mutant BRAF^{V600E} melanoma cells are growth promoted by low dose PLX-4720. (a) Images of Colo829 cells treated with the indicated doses of the mutant BRAF inhibitor PLX-4720 (IncuCyte Zoom 10x magnification). **(b)** Western blots of lysates from Colo829 cells treated with 62.5nM PLX-4720 for the times indicated. **(c)** A375(M2) colony formation assay in 10cm dishes in the presence of solvent control, SB-431542 (10μM), PLX-4720 (31.25nM) or both drugs.

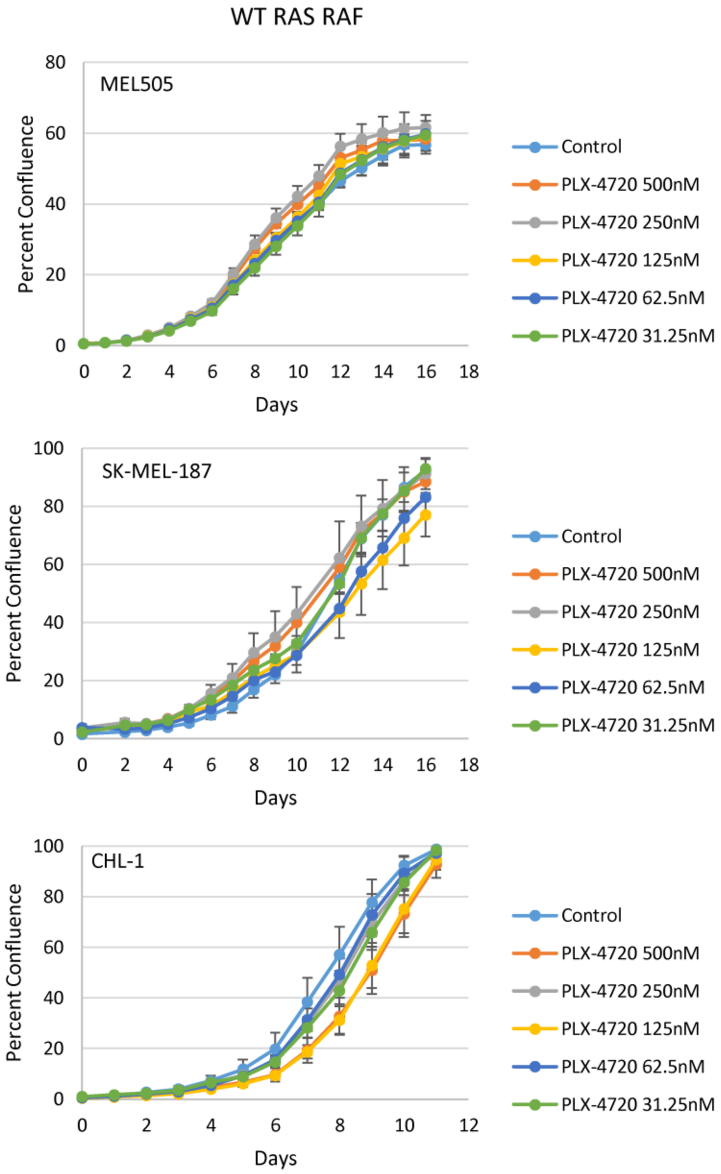

Supplementary Figure 7. Low dose BRAF inhibitor (PLX-4720) does not promote growth of wild type RAS/RAF melanoma cells. Cell proliferation assays were carried out by live cell imaging (IncuCyte Zoom). Wild type (WT) BRAF and RAS cell lines: MEL505 cells (400 cells/well of 96-well plate), SK-MEL-187 (200 cells/well of 96-well plate) and CHL-1 (100 cells/well of 96-well plate) were seeded overnight and treated with PLX-4720 at the concentrations indicated. The mean percent confluence (± SEM) are shown (data were generated from 12 fields from three independent wells from a representative experiment).

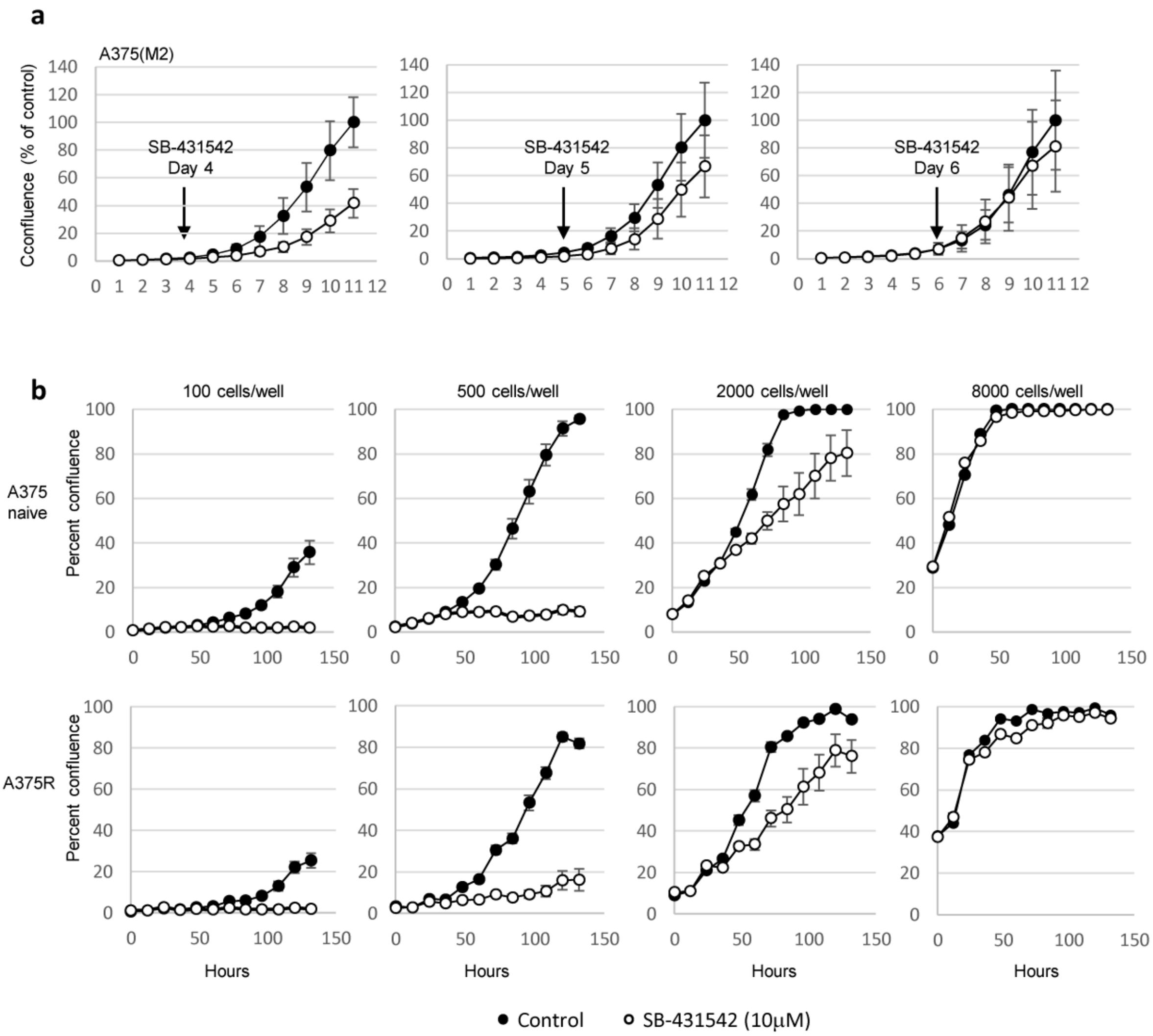

Supplementary Figure 8. TGFβR1 inhibitors are effective during 'clonogenic stress' when cells are seeded at low density (a) A375(M2) cells were seeded at low cell density. SB-431542 (10μM) was added at increasingly later stages during the lag phase of growth. **(b)** A375 naïve and A375R PLX-4720 resistant cells were seeded at increasing cell densities as indicated and monitored for proliferation over time in the IncuCyte in control conditions (closed circles) and following treatment with 10μM SB-431542 (open circles).

Table S1. Growth conditions and source of cell lines.

Cell line	RAS family mutation	Description	Growth conditions*	Source
Melan-a		Murine immortalised melanocytes	RPMI 1640 + 10ng/ml cholera toxin + 120ng/ml PMA	Richard Marais
Melan-a wt B-Raf		Murine immortalised melanocytes	RPMI 1640 + 10ng/ml cholera toxin + 120ng/ml PMA	Richard Marais
Melan-a B-RafV600E		Murine immortalised melanocytes	RPMI 1640	Richard Marais
A375	BRAF	Human malignant melanoma	DMEM	Richard Marais
A375R	BRAF	Human melanoma, vemurafenib resistant	DMEM + 1μM PLX-4720	Richard Marais
A375(M2)	BRAF	Human malignant melanoma	DMEM	Richard Marais
Colo-829	BRAF	Human malignant melanoma	RPMI 1640	Richard Marais
HT29	BRAF	Human colorectal adenocarcinoma	DMEM	Simon Cook
WM266-4	BRAF	Human metastatic melanoma	DMEM	Richard Marais
Colo 205	BRAF	Human colorectal adenocarcinoma	RPMI 1640	Richard Marais
RKO	BRAF	Human colon carcinoma	DMEM	Kevin Ryan
WM35	BRAF	Human malignant melanoma	RPMI 1640	Richard Marais
VM-CUB-1	HRAS	Human bladder carcinoma	DMEM	Wolfgang Schulz
T-24	HRAS	Human bladder carcinoma	DMEM	Wolfgang Schulz
KNS-62	HRAS	Human lung squamous cell carcinoma	RPMI 1640	Holger Kalthoff
Hs578t	HRAS	Human breast carcinoma	DMEM	Mina Bissel
AsPC-1	KRAS	Human pancreatic ductal carcinoma	RPMI 1640	Jeff Evans
LS-174T	KRAS	Human colorectal adenocarcinoma	DMEM	Simon Cook
DLD-1	KRAS	Human colorectal adenocarcinoma	RPMI 1640	Simon Cook
A549	KRAS	Human lung carcinoma	DMEM	ATCC
SK-CO-1	KRAS	Human colon adenocarcinoma	DMEM	Simon Cook
UM-UC-3	KRAS	Human bladder carcinoma	DMEM	Wolfgang Schulz
MIA-PaCa-2	KRAS	Human pancreatic ductal carcinoma	DMEM	Jeff Evans
PANC-1	KRAS	Human pancreatic ductal carcinoma	DMEM	Jeff Evans
LoVo	KRAS	Human colorectal adenocarcinoma	Hams F12	CRUK cell services
SW620	KRAS	Human colon adenocarcinoma	DMEM	Simon Cook
MDA-MB-231	KRAS	Human breast carcinoma	DMEM	CRUK cell services
SK-MEL-2	NRAS	Human malignant melanoma	DMEM	Richard Marais
SK-MEL-119	NRAS	Human malignant melanoma	DMEM	Norman Sharpless
SK-MEL-147	NRAS	Human malignant melanoma	DMEM	Norman Sharpless
SK-MEL-173	NRAS	Human malignant melanoma	DMEM	Norman Sharpless
RPMI-8332	WT	Human malignant melanoma	RPMI 1640	Norman Sharpless
MDA-MB-361	WT	Human breast carcinoma	DMEM	CRUK cell services
PC-3	WT	Human prostate adenocarcinoma	DMEM	Marene Landstrom
SK-MEL-23	WT	Human melanoma	DMEM	Norman Sharpless
SK-MEL-187	WT	Human melanoma	DMEM	Norman Sharpless
Mel-505	WT	Human malignant melanoma	RPMI 1640	Norman Sharpless
CHL-1	WT	Human malignant melanoma	DMEM	Richard Marais
A431	WT	Human skin squamous cell carcinoma	DMEM	CRUK cell services
Patient#2	BRAF	Human stage 4 melanoma; vemurafenib 3 mnths; PR	RPMI 1640 + 1μM PLX-4720	Richard Marais. Ref 18
Patient#5	BRAF	Human stage 4 melanoma; vemurafenib 2 mnths; PD	RPMI 1640 + 1μM PLX-4720	Richard Marais. Ref 18
Patient#35	BRAF	Human stage 4 melanoma; vemurafenib 3 mnths; PR	RPMI 1640 + 1μM PLX-4720	Richard Marais
Patient#1	BRAF	Human stage 3 melanoma no treatment (naïve)	RPMI 1640	Richard Marais. Ref 18

All media was also supplemented with 10% FBS, glutamine and antibiotics.

PR, partial response; PD, progressive disease; WT, wild type;

Information regarding the mutational status of cell lines used can be found at the Wellcome Trust Sanger Centre COSMIC cell lines project website http://cancer.sanger.ac.uk/cell_lines

Table S2. TGFβ1 production by tumour cell lines.

Cell line	TGFβ1 (pg per $1x10^5$ cells/hour	n	Effect of SB-431542
A375	33.1 ± 2.7	5	inhibited
Colo-829	86.57 ± 2.9	5	inhibited
HT29	8.2 ± 1.1	3	inhibited
WM266-4	80.4 ± 1.7	5	inhibited
WM35	49.7 ± 2.9	5	inhibited
Hs578T	99.2 ± 5.26	3	promoted
PANC-1	59.75 ± 1.33	3	no effect
SK-MEL-147	5.66 ± 0.14	3	promoted
SK-MEL-173	14.66 ± 0.14	3	promoted
CHL-1	36.9 ± 3.2	5	promoted

Table S2. The amount of TGFβ1 produced by the indicated cell lines seeded at 50% confluency and in log phase of growth was analysed by ELISA and is expressed as the amount (pg) of TGFβ1 produced by $1x10^5$ cells/hour. The number of measurements of TGFβ1 production is shown (n).
